# Pumping approximately integrable systems

**DOI:** 10.1038/ncomms15767

**Published:** 2017-06-09

**Authors:** Florian Lange, Zala Lenarčič, Achim Rosch

**Affiliations:** 1 Institute for Theoretical Physics, University of Cologne, Zülpicher Straße 77a, D-50937 Cologne, Germany

## Abstract

Weak perturbations can drive an interacting many-particle system far from its initial equilibrium state if one is able to pump into degrees of freedom approximately protected by conservation laws. This concept has for example been used to realize Bose–Einstein condensates of photons, magnons and excitons. Integrable quantum systems, like the one-dimensional Heisenberg model, are characterized by an infinite set of conservation laws. Here, we develop a theory of weakly driven integrable systems and show that pumping can induce large spin or heat currents even in the presence of integrability breaking perturbations, since it activates local and quasi-local approximate conserved quantities. The resulting steady state is qualitatively captured by a truncated generalized Gibbs ensemble with Lagrange parameters that depend on the structure but not on the overall amplitude of perturbations nor the initial state. We suggest to use spin-chain materials driven by terahertz radiation to realize integrability-based spin and heat pumps.

## Abstract (web summary)

Integrable models have an infinite number of conserved quantities but most realizations suffer from integrability breaking perturbations. Here the authors show that weakly driving such a system by periodic perturbations leads to large nonlinear responses governed by the approximate conservation laws.

A simple classical example for a weakly driven system is a well-insulated greenhouse. Due to the approximate conservation of the energy within the greenhouse, even weak sunlight can lead to high temperatures in its interior, which can be computed from the simple rate equation for the energy transfer. Similarly, large spin accumulation can be achieved in systems with approximate spin conservation^1^. Using approximate conservation of the number of photons, magnons or exciton polaritons, one can use pumping by light to reach densities, which allow for the realization of Bose–Einstein condensates^2^,^3^,^4^. Number-conserving collisions induce a quasi-equilibrium state in these systems, which can be efficiently described by introducing a chemical potential whose value is determined by balancing pumping and decay processes. Related theoretical approaches that describe electron-phonon systems far from equilibrium are so-called two-temperature models^5^: here one uses that the energy of the electrons and phonons are approximately separately conserved to introduce two different temperatures for the subsystems.

Integrable many-particle systems, like the one-dimensional (1D) fermionic Hubbard model or the XXZ Heisenberg model, are described by an infinite number of (local or quasi-local) conservation laws^6^,^7^,^8^,^9^,^10^,^11^. In closed integrable systems those prevent the equilibration into a simple thermal state, for example, after a sudden change of parameters. Instead the system can be described by a generalized Gibbs ensemble (GGE)^12^,^13^,^14^,^15^,^16^,^17^,^18^,^19^,^20^,^21^


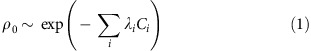


where *C*_*i*_ are the conserved quantities and *λ*_*i*_ the corresponding Lagrange parameters. It has also been shown experimentally^22^ that GGEs for a Lieb–Liniger model can provide highly accurate descriptions of interacting bosons in 1D.

Many materials are described with high accuracy by integrable models^23^, however, weak integrability breaking terms and the coupling to thermal phonons imply that in equilibrium these systems are described by simple thermal states, 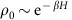, instead of GGEs. The proximity to the integrable point and the presence of approximate conservation laws leads to enhanced spin or heat conductivities (within linear-response theory)^24^,^25^,^26^ and also to a slow relaxation after a quantum quench (via GGE-prethermalization) towards the equilibrium state^27^.

We will show that—as in the greenhouse example, see Fig. 1—such an approximately integrable system can be driven far from its thermal equilibrium by weak perturbations arising, for example, from a driving periodic in time or from coupling to a non-thermal bath. To balance the constant heating due to driving the system has to be weakly open, for example, by coupling to a phonon bath. As we will demonstrate this mechanism can be used for example to create large spin and heat currents. Besides the quasi-1D systems considered by us, also approximately many-body localized systems are characterized by infinitely many approximate conservation laws which may lead to a strong response to driving^28^,^29^.

## Results

### Weakly driven system

We consider an interacting many-body system that is approximately described by Hamiltonian *H*_0_ and characterized by a finite or infinite number of (quasi-)local conserved quantities *C*_*i*_, [*H*_0_, *C*_*i*_]=0, one of them being *H*_0_. Energy and other conservations are weakly broken by coupling to thermal or non-thermal baths and/or perturbations periodic in time. For simplicity, we assume periodic boundary conditions and a (discreet) translational invariance. We describe the system with density matrix *ρ* whose dynamics is governed by the Liouvillian super-operator 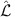,


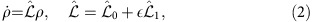


where 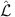 can be split into the dominant unitary Hamiltonian evolution 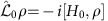 and perturbation 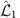 of strength 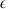. We are interested in the limit of small 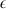 for *t*→∞, where a unique (Floquet) steady state *ρ*_∞_ is obtained. The general structure of perturbation theory in this case has, for example, been discussed in refs 30, 31, 32. In this limit, *ρ*_∞_ can be approximated by 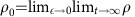 with 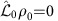 according to equation (2). We assume and later support numerically that *ρ*_0_ is approximately described by a GGE, see equation (1).

Here it is essential to note that—as in the greenhouse example discussed above—the parameters *λ*_*i*_ are not determined by the initial state but by the form of the weak perturbations 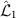. Our central goal is to compute the *λ*_*i*_. We first discuss the case of Lindblad dynamics, where perturbation theory linear in 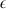 can be used, and then focus on Hamiltonian dynamics where we have to consider 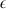
^2^ contributions.

### Markovian perturbation

Within the Markovian approximation one can use the Lindblad form for 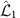 (ref. 33). Note that Lindblad dynamics is considered here mainly for pedagogical purposes (formulas are simpler) while no Lindblad approximation is used for the models studied below. The coefficients *λ*_*i*_ that fix the GGE are determined from the condition that the change of the approximately conserved quantities has to vanish in the steady state


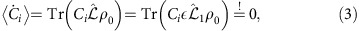


where we used that 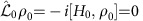. Relation (3) yields a set of coupled equations for *λ*_*i*_, where the number of equations is equal to the number of conserved quantities. We define the super-projector 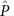 onto the tangential space of GGE density matrix,


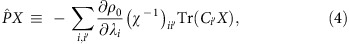


using *χ*_*ii*′_=−Tr(*C*_*i*_*∂ρ*_0_/*∂λ*_*i*′_). Then the conditions for *ρ*_0_ can be compactly written as


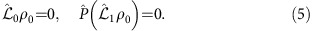


This equation can also be derived by considering higher order perturbations in 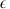, see Methods for details.

### Hamiltonian perturbation

For Hamiltonian dynamics 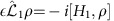, where *H*_1_ may be a sum of several integrability breaking perturbations. Perturbation theory linear in 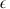 vanishes, 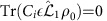 for all *λ*_*i*_. Therefore one has to expand to order 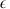
^2^ and equation (5) is replaced by


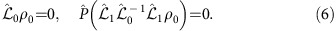


Since 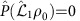, 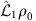 is not in the kernel of 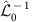. For periodic driving this equation has to be interpreted within the Floquet formalism, see Methods.

### Model

As discussed in the introduction, our goal is to describe a situation which can be realized experimentally in spin-chain materials driven by lasers operating in the terahertz regime. We assume that spin chains are approximately described by a spin-1/2 XXZ Heisenberg model, possibly in the presence of an external magnetic field *B*,


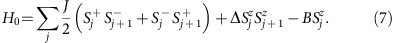


**Figure 1:**
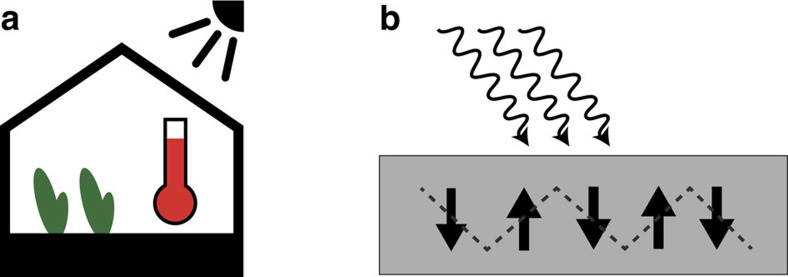
Greenhouse principle. (**a**) A well-insulated greenhouse exposed to sunshine can heat up significantly since energy within it is approximately conserved. (**b**) As the heat current in spin-chain materials is approximately conserved even weak terahertz radiation can induce large heat current. Material candidates must have appropriate crystal structure, schematically denoted by dashed lines indicating alternating chemical bonds.

The system is driven out of equilibrium by a weak (integrability breaking) time-dependent perturbation


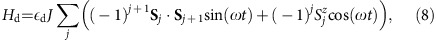


with driving frequency *ω*. This specific term has been chosen because it can induce heat and spin currents (as can be shown by a symmetry analysis), and because it can be realized experimentally. Such staggered exchange couplings and staggered magnetic fields arise naturally in certain compounds with (at least) two magnetic atoms per unit cell when coupled to uniform electric and magnetic fields, respectively^34^,^35^,^36^,^37^. See Fig. 1 for a schematic drawing of such a compound and Methods for concrete experimental suggestions. Therefore *H*_d_ can be realized by shining a laser (typically at terahertz frequencies) onto the sample. In this case 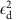 is proportional to the laser power. Note that for *T*=0 and *B*=0 in the adiabatic limit, *ω*→0, equations (7) and (8) realize an adiabatic Thouless pump, where per pumping cycle one spin is transported by one unit cell^38^. We will be interested in the opposite regime of large *ω* and large (effective) temperatures.

Formally, the periodic perturbation *H*_d_ would drive the system to infinite temperature^39^,^40^,^41^,^42^ (up to remaining conservation laws^43^, possibly through a prethermal-like regime^44^). In a solid state experiment this is prohibited by the coupling to phonons and, ultimately, to the thermal environment of the experimental set-up. We mimic this effect by coupling the spin system to a bath of Einstein phonons, 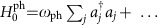, where dots stand for the couplings to further reservoirs which guarantee that the phonon system is kept at fixed temperature *T*_ph_, 
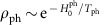
. See Methods for details on finite size calculation using a broadened distribution of phonon energies. The (weak) coupling to the spin system is described by


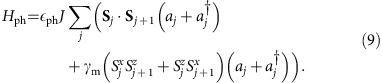


To obtain a unique steady state it is essential to break all symmetries, including the *S*^*z*^ conservation. Relativistic effects which relax *S*^*z*^ are mimicked by *γ*_m_ in our approach. We expect 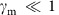 in materials without heavy elements. For simplicity, we set *γ*_m_=1 within our numerics as this is found to minimize finite size effects, without a qualitative influence on the results. Besides phonons also other integrability breaking perturbations exist in real materials, including defects, which typically dominate at the lowest temperatures. For high temperatures of the order of *J* (relevant for the considered set-up) it is realistic to assume that phonon coupling dominates.

In the presence of a periodic perturbation, equation (8), in the long-time limit the density matrix is changing periodically, *ρ*(*t*→∞)=∑_*n*_e^−*iωnt*^*ρ*^(*n*)^ with 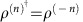, 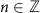. Within the Floquet formalism one therefore promotes the steady-state density matrix to a vector and Liouville operator to a matrix, see Methods. For weak driving, 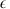
_d_→0, only the *n*=0 sector remains and the GGE ansatz, equation (1), simply reads 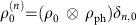, where we included also the phonon density matrix, see above.

### Steady state

We will use two different approaches to determine an approximate solution for the steady-state density matrix. First, we will parametrize *ρ*_0_, equation (1), with a small number of (quasi-)local conserved quantities, *C*_*i*_, *i*=1, …, *N*_C_. In an alternative approach, feasible for small systems, we take all conserved quantities into account: local and non-local, commuting and non-commuting. While the second approach is formally exact in the limit 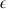
_d_,
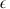
_ph_→0, the first one is, perhaps, more intuitive and can be computed for larger system sizes.

For the XXZ Heisenberg model an infinite set of mutually commuting local conserved quantities *C*_*i*_ is known, see Methods. *C*_1_ is the total spin 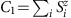 and *C*_2_=*H*_XXZ_. Importantly, *C*_3_ is the heat current^45^ *C*_3_=*J*_H_(*B*=0). In addition there also exist (infinite) sets of quasi-local commuting conserved quantities^8^,^9^,^10^. As shown in refs 8, 46 the spin-reversal parity-odd family has an overlap with the spin current *J*_S_ at Δ<*J*. Therefore both heat and spin current could show a large response to a weak perturbation. For our analysis, we choose three or five (*N*_C_=4, *N*_C_=6) most local conserved quantities *C*_*i*_, *i*=1, …, *N*_C_−1. From the quasi-local sets, we include as a single (effective) operator the conserved part of spin current *J*_S_^c^, computed numerically^25^,^47^. For details see Methods. In the presence of an external magnetic field, equation (7), the heat current also has, in addition to *C*_3_, a spin current component, *J*_H_=*C*_3_−*BJ*_S_ (ref. 45).

**Figure 2:**
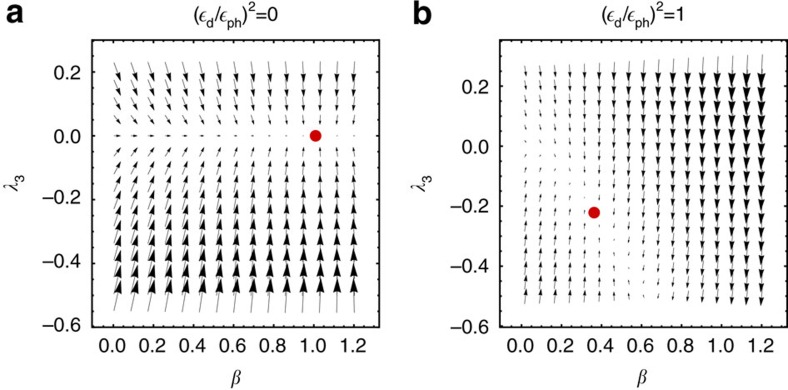
Force field with thermal and non-thermal steady state. Effective force **F** in the space of Lagrange parameters (*β*, *λ*_3_) using 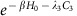 as an ansatz for the generalized Gibbs ensemble. Parameters: *J*=Δ=−*B*=*ω*=*ω*_ph_=*T*_ph_. Lagrange parameters (*β*, *λ*_3_) are plotted in units 1/*J* and 1/*J*^2^, respectively. (**a**) In the absence of an external driving, 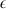
_d_=0, the stable fixed point (red dot) is given by the thermal ensemble, *β*=1/*T*_ph_, *λ*_3_=0. (**b**) When the system is driven by *H*_d_ (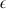
_d_=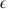
_ph_), it heats up and *λ*_3_ becomes finite.

For the visualization of our results it is useful to define generalized forces *F*_*i*_ in the space of Lagrange parameters by rewriting 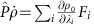 such that 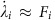,


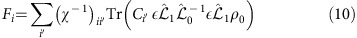


computed using exact diagonalization, see Methods. The vector **F** is a function of the Lagrange parameters *λ*_*i*_, which points into the direction of the steady-state stable fixed point obtained from *F*_*i*_=0. In the absence of driving (Fig. 2a) one obtains the expected thermal state with *T*=*T*_ph_ while all other Lagrange parameters *λ*_*i*_ vanish. For finite driving the GGE is activated and the *λ*_*i*_ become finite (Fig. 2b). To obtain the steady state, we solve *χ***F**=0 using Newton's method.

For the second approach, performed on small *N*-site systems, we first numerically construct a basis in the set of all (local and non-local) conserved operators, 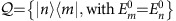, where 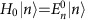. Due to degeneracies we find (for finite *B* and Δ≠*J*) about 2·2^*N*^ elements 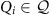. In the limit 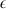
_d_, 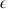
_ph_→0 the steady-state density matrix *ρ*_∞_ has to fulfil 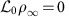 and therefore can be exactly written as a linear combination of the *Q*_*i*_, 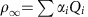. Using equation (6), we therefore find that the steady-state density matrix for 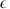
_d_, 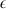
_ph_→0 is exactly given by the unique eigenvector with eigenvalue zero of the matrix


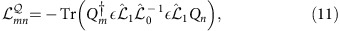


where 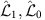 are Floquet matrices, see Methods. Note that only the relative 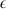
_d_/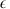
_ph_ and not the absolute strength of perturbations determine *ρ*_0_, as can be seen by dividing the equations *χ***F**=0 or 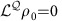 by 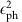.

**Figure 3:**
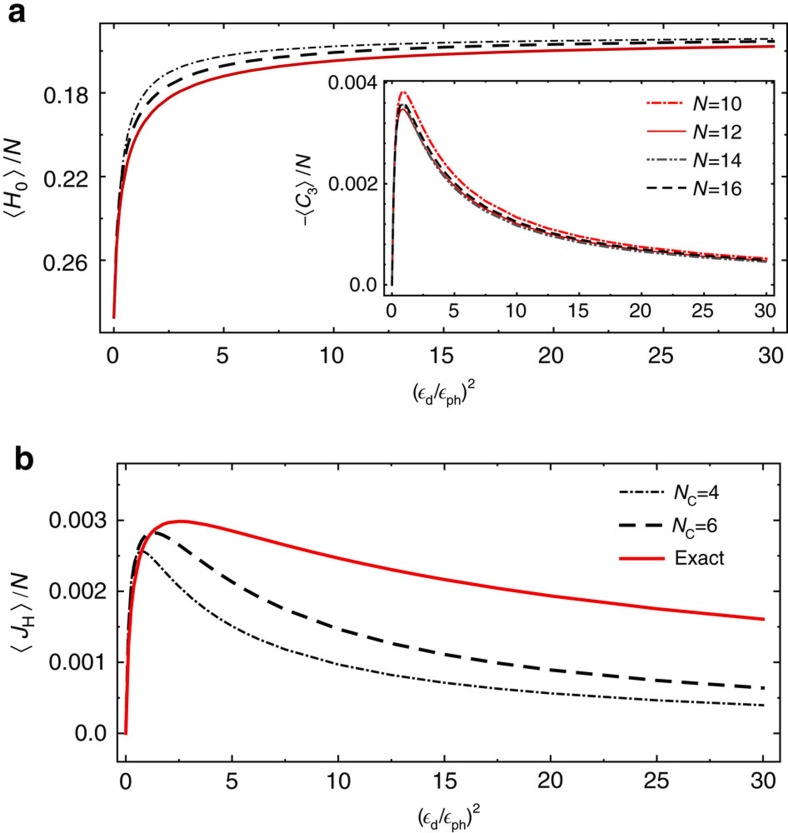
Steady-state expectation values of conserved quantities. Expectation values of (**a**) energy and (**b**) heat current densities for a weakly driven spin chain, 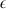
_d_, 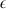
_ph_→0, as functions of the ratio of driving strength 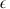
_d_ and phonon coupling 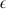
_ph_. Red solid lines: exact result taking into account all 7,969 conservation laws of a system of *N*=12 sites. (**a**) For the energy accurate results are already obtained with a GGE ensemble based on *N*_C_=4 (dot-dashed lines) or *N*_C_=6 (dashed lines) conserved quantities. (**b**) Also the heat current *J*_H_=*C*_3_−*BJ*_S_ is qualitatively well described by the GGE ensemble but quantitative deviations are larger. Inset: Finite size analysis for (local) *C*_3_ based on GGE ensemble with *N*_C_=6 conserved quantities. Parameters: *J*=1, Δ=0.8, *B*=−1.0, *ω*=1.6 *ω*_ph_, *ω*_ph_=*T*_ph_=1.

In Fig. 3, we show the expectation value of the energy and of the heat current densities as functions of 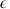
_d_/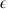
_ph_ taking into account *N*_C_=4, *N*_C_=6, and all conserved quantities. The energy density expectation value is already obtained with good accuracy for *N*_C_=4 and even better for *N*_C_=6. The heat current vanishes both in thermal equilibrium, 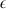
_d_→0, and for 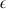
_ph_→0, where the system is described by an infinite temperature state with finite magnetization, 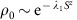 and 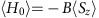. It takes its largest value for 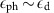. For the currents a description in terms of *N*_C_=4 or 6 is qualitatively but not quantitatively accurate. Our study strongly suggests that further quasi-local conserved quantities contribute, as discussed in quench protocols^15^,^16^,^17^, see also ref. 25. For the chosen parameters our results depend only weakly on the system size *N*, see inset of Fig. 3. System size analysis is performed for *N*_C_=6 since the solution based on all conservations cannot be obtained for larger systems.

**Figure 4:**
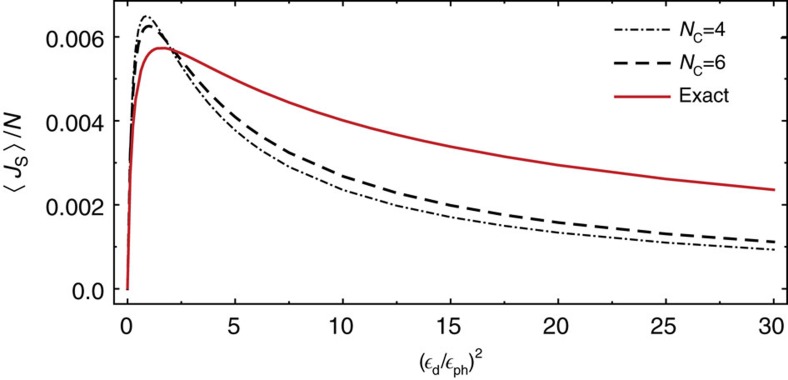
Spin current in the steady state. For vanishing magnetic field a spin current (but no heat current) is generated within our model for finite ratios of 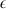
_d_/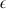
_ph_. The expectation value of spin current density is again maximal for 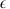
_d_/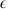
_ph_≈1. Parameters: *J*=1, Δ=0.8, *ω*=1.6 *ω*_ph_,*ω*_ph_=*T*_ph_=1, *N*=12.

Our set-up can also be used to create spin currents. Whilst, by symmetry (bond-centered rotation in real and spin space by *π* around *y* axis), a finite external field *B* is needed to obtain a finite heat current, this is not the case for the spin current. Figure 4 displays the spin current density as a function of 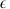
_d_/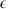
_ph_ for *B*=0. Qualitatively one obtains a behaviour rather similar to the results for the heat current shown in Fig. 3 with a maximum in the spin current for 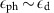.

**Figure 5:**
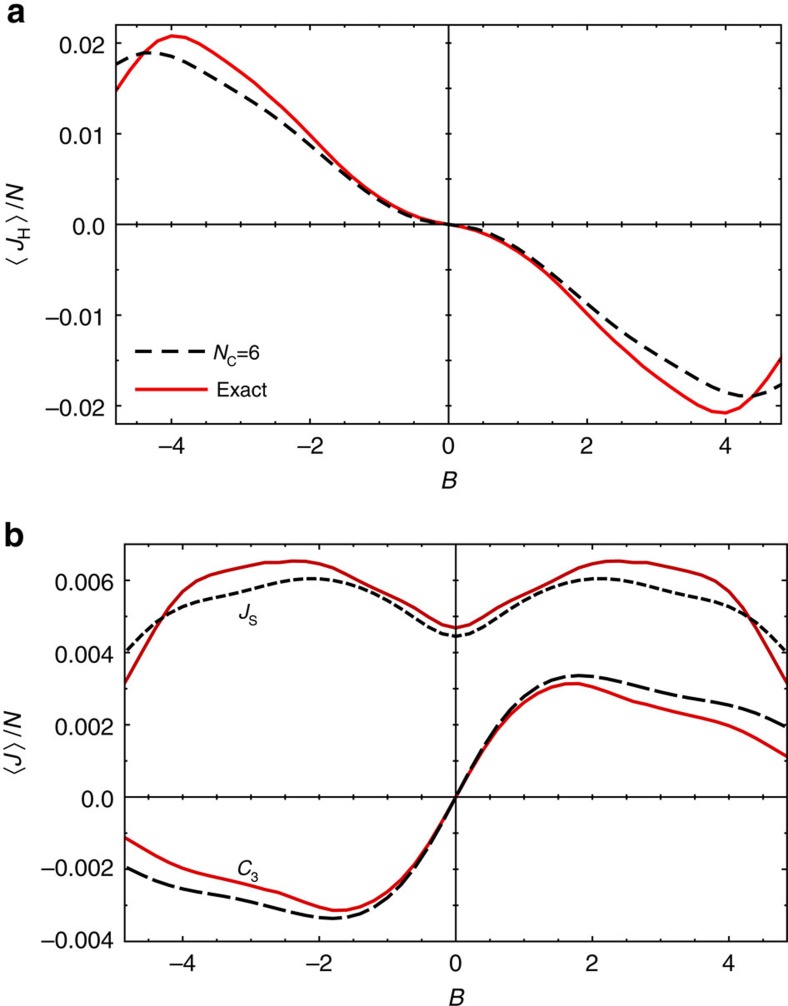
Currents in the steady state at different magnetic field. (**a**) Heat current *J*_H_, (**b**) spin current *J*_S_, and *C*_3_ densities as a function of external magnetic field *B* obtained from a GGE ensemble with *N*_C_=6 conserved quantities (dashed) or from an exact calculation (solid) including all conservations. Parameters: 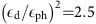, *J*=1, Δ=0.8, *ω*=1.6 *ω*_ph_, *ω*_ph_=*T*_ph_=1, *N*=12.

The external magnetic field *B* is a parameter which can easily be tuned experimentally. Figure 5 shows heat and spin current densities as a function of external magnetic field *B* for 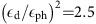. Note that the sign of the magnetic field determines the sign of the heat current 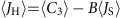. All main features of the B-dependence are semi-quantitatively reproduced by the truncated GGE with *N*_C_=6. For very large magnetic fields the convergence to the steady-state fixed point becomes slow as transitions rates connecting sectors with different magnetization are strongly suppressed, see Methods for further details.

## Discussion

We have demonstrated that driving approximately integrable systems activates and pumps into approximately conserved quantities. Perhaps the most simple experimental set-up to measure the pumping effect predicted in this work, is to use a terahertz laser that excites a spin-chain material like Cu-benzoate where by symmetry staggered terms of the form (8) are expected^34^,^35^. As a consequence of the induced heat currents it is anticipated that the system cools down on one side while it heats upon the other. The direction of the effect can be controlled either by changing the direction of the laser beam or the sign of the external magnetic field *B*.

For the chosen parameters the spin and heat currents expressed in dimensionless units appear to be rather small, of the order of 10^−3^. While these values can definitely be increased by tuning parameters, for example the external magnetic field, it is important to note that the currents are actually quite large compared to the typical heat or spin currents obtained in bulk materials. To create a heat current of similar size in a good heat conductor like Cu (assuming 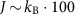 K, 5 Å for the distance of the spin chains, and *κ*^Cu^≈400 Wm^−1^ K^−1^) one would need a temperature gradient of several 10^5^ Km^−1^. Similarly, to create a (transversal) spin current of comparable size in a heavy element like Pt using the spin Hall effect (assuming *ρ*^Pt^≈10 μΩ cm and 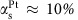 for the spin Hall angle^48^) one needs electric fields of the order of 10^4^ V m^−1^ or sizable current densities of the order of 10^11^ Am^−2^. These numbers are even more remarkable when one takes into account that the electron densities in Cu or Pt are at least an order of magnitude higher than the spin density for spin chains with a distance of 5 Å.

While our study has focused on the steady state, it is instructive to discuss the relevant timescales for its buildup. For this argument, we consider a quench where at time *t*=0 an initial state is perturbed both by the integrable part of the Hamiltonian and by small non-integrable perturbations. At short times of the order of several 1/*J* the initial state will prethermalize^27^,^49^,^50^,^51^,^52^ into a GGE, where the values of the conserved quantities, 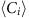, are set by the initial conditions (with small corrections from the perturbations^52^,^53^). Further time evolution can be approximately described by a GGE with time-dependent Lagrange parameters. Their time-dependence is determined by perturbations which assert forces 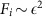, such that d*λ*_*i*_/d*t*≈*F*_*i*_. Governed by the perturbations the system will loose the memory of its initial condition on a timescale of order 1/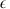
^2^ and relax to the steady state (obtained from *F*_*i*_=0) which is, in general, completely unrelated to the prethermalized state. Note that the same approach predicts ordinary thermalization in the absence of external driving.

Our results suggest that the concept of generalized GGEs has a much broader range of application than previously anticipated, now extended to open systems where symmetries are not exact and integrability is weakly broken. A truncated GGE proved to be useful for qualitative description, however, it showed quantitative discrepancies most probably due to disregarded quasi-local conserved quantities, as observed already in quench protocols^15^,^16^. We are planning a future study tailored to address this issue systematically. It would be interesting to develop integrability-based methods similar to the quench-action approach^15^,^16^,^54^,^55^ to treat such situations.

Most important for applications is that the integrability is not required to be realized exactly but only approximately. Efficient pumping requires only that the pumping rates are of the same order of magnitude as the loss rates arising from integrability breaking terms. Especially the integrability-based creation of large spin currents could find its application in future spintronics devices.

## Methods

### Perturbing around *ρ*_0_

The central equations (5) or (6), 26, used to determine the density matrix *ρ*_0_ in the limit 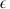
→0, have to be consistent and can also even be derived by considering perturbations around *ρ*_0_, *ρ*_∞_=*ρ*_0_+*δρ*.

First, the leading *δρ* correction to 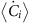, equation (3), arising from 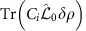 which is nominally of the same order as 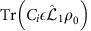 vanishes trivially as Tr(*C*_*i*_[*H*_0_, *δρ*])=Tr(*δρ*[*C*_*i*_, *H*_0_])=0.

For arbitrary *ρ*_0_, *δρ* is exactly given by 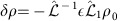, where 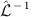 is a short-hand notation for 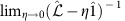 with the infinitesimal regularizer *η*. The correct expansion point *ρ*_0_ is found if 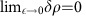. Below we show that for the projection operator 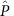, equation (4),


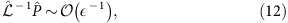


which would yield 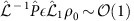. This contradicts our perturbative approach unless 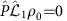, as set by our condition equation (5).

Equation (12) is a consequence of the fact that 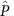 projects onto the tangential space to GGE density matrix. In this space 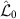 vanishes by definition, 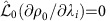, and 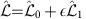 is therefore of order 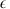. Technically, this can be seen by using the general relation


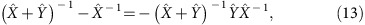


for


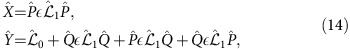


with 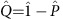 and 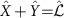. Then


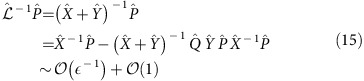


The second term is *O*(1) as 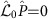 and therefore 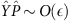. The divergence of 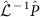 for 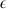
→0 can be directly related to the fact that integrable systems are characterized by infinite conductivities (finite Drude weights) at finite temperatures^56^ as can, for example, be seen^24^ within the memory matrix formalism^57^.

All arguments given above can be generalized to situations where leading corrections arise from second-order perturbation theory in which case one obtains equation (6) instead of equation (5).

### Staggered hopping and magnetic field modulation

Sizable staggered g-tensors leading to staggered B-fields have been observed in a number of different compounds^34^,^35^,^36^,^37^. Similarly an external electric field will distort the crystalline structure in these materials, leading to staggered exchange couplings linear in homogeneous electric fields. An example of such a material is Cu-benzoate^34^ with the above modulations allowed by symmetry for electric (magnetic) fields applied in the 010 (001) crystallographic direction. In this system the staggered g-tensor has been measured to be ∼0.08 (ref. 35), the size of the staggered exchange coupling is unknown. For simplicity, we assume in equation (8) that the two staggered terms are of the same size.

### Conservation laws of the XXZ Heisenberg model

An infinite set of local conserved quantities *C*_*i*_ of the Heisenberg model *H*_XXZ_=*H*_0_(*B*=0) can be obtained using the boost operator 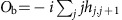 (where *H*_XXZ_=∑_*j*_ *h*_*j*,*j*+1_) from the recursion relation [*O*_b_, *C*_*i*_]=*C*_*i*+1_ for *i*>1 with 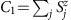, *C*_2_=*H*_XXZ_ (ref. 7). In general, *C*_*i*_ are operators involving maximally *i* neighbouring sites. Importantly, *C*_3_ in the absence of external magnetic field equals the heat current


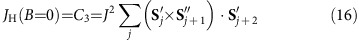


with rescaled spin operators 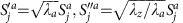 for *λ*_*z*_=Δ/*J*, *λ*_*x*_=*λ*_*y*_=1. In the presence of external magnetic field, equation (7), heat current has in addition to *C*_3_ also a spin current component,


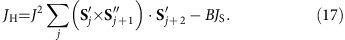


As understood recently there also exist families of quasi-local conserved quantities^8^,^9^,^10^, which are mostly disregarded in our study with the exception of a spin-reversal parity-odd operator, *J*_S_^c^. The latter is constructed as the conserved part of the spin current operator *J*_S_,


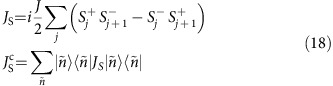


where 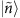 are simultaneous eigenstates of the *C*_*i*_. Since it is known that the spin current has an overlap with the quasi-local family^45^ for Δ<*J*, the conserved *J*_S_^c^ contains quasi-local components (and, possibly, non-local components not contributing in the thermodynamic limit).

### Floquet formulation

For a periodically driven system described by 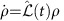 with 
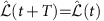
 the density matrix changes periodically in the long-time limit. Therefore it is useful to split it into Floquet components,


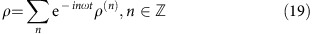


with 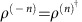 and *ω*=2*π*/*T*. The Floquet components are combined into the vector 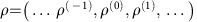. The Liouvillian is promoted to a (static) matrix 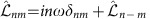 with 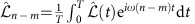. Using this notation, all results obtained for static Liouvillian super-operators directly translate to the time-periodic case. Within our set-up *H*_0_, all approximate conservation laws *C*_*i*_ and the GGE density matrix *ρ*_0_ are static and therefore the projection operator 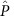, equation (4), projects onto the *n*=0 Floquet sector only. The steady-state condition, equation (6), thus means that the approximately conserved quantities do not grow after averaging over an oscillation period. To second order in 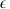
_d_ only transitions from the *n*=0 to the *n*=±1 Floquet sector and back contribute to equations (6) or (10), 46 as 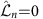 for 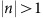.

For the generalized force due to the periodic driving, we obtain from (10)


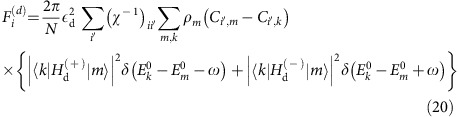


where we used *H*_0_ eigenstates 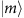 with 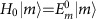, matrix elements 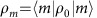, 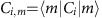, and the notation 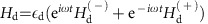. Note that equation (20) contains—as expected—transition rates well-known from Fermi's golden rule. Equation (20) is evaluated for finite systems of size *N* by replacing the *δ* function by a Lorentzian (1/*π*)*η*/(*ω*^2^+*η*^2^) (*η*=0.1*J* for *N*=12).

Equation (20) is only valid for situations where all conservation laws commute with each other, with 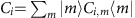, see below for a brief discussion of the non-commuting case.

### Phonon coupling

As written in the main text, we assume that the phonon system always remains at equilibrium, 
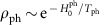
. Using equation (10), after tracing over phonons, we obtain for the generalized force


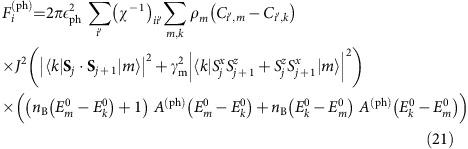


where 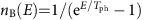 is the equilibrium Bose distribution evaluated at the temperature *T*_ph_ and *A*^(ph)^(*ω*) is the phonon spectral function. For our finite size calculation, we broaden the spectral function of the Einstein phonons using 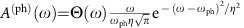. This choice of broadening ensures detailed balance relations (necessary to obtain a thermal state in the absence of driving) and the positivity of phonon frequencies (necessary for stability). For all plots we use *η*=0.4*J*. However, we have checked that similar results are obtained, for example, for *η*=0.1*J* for magnetic fields up to 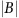
=2*J*. For larger fields *η*=0.1*J* does not provide a sufficient amount of relaxation between sectors with different magnetization and convergence becomes slow and unstable. For *η*=0.4*J* larger fields, 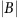
≲5*J*, can be reached.

### Implementation of non-commuting conservation laws

As discussed in the main text, a complete basis of all non-local commuting or non-commuting conserved quantities is given by 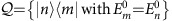 which solve the equation 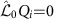 for 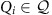. Using the exact eigenstates of *H*_0_ it is straightforward to evaluate equation (11), where we use for our finite size calculations the broadening procedures described above. As a technical detail, we note that, when one follows this procedure, one has to evaluate in the phonon sectors integrals of the type 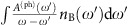 numerically. For efficient evaluations, we use interpolating functions for these integrals.

### GGE estimation for other conserved quantities

**Figure 6:**
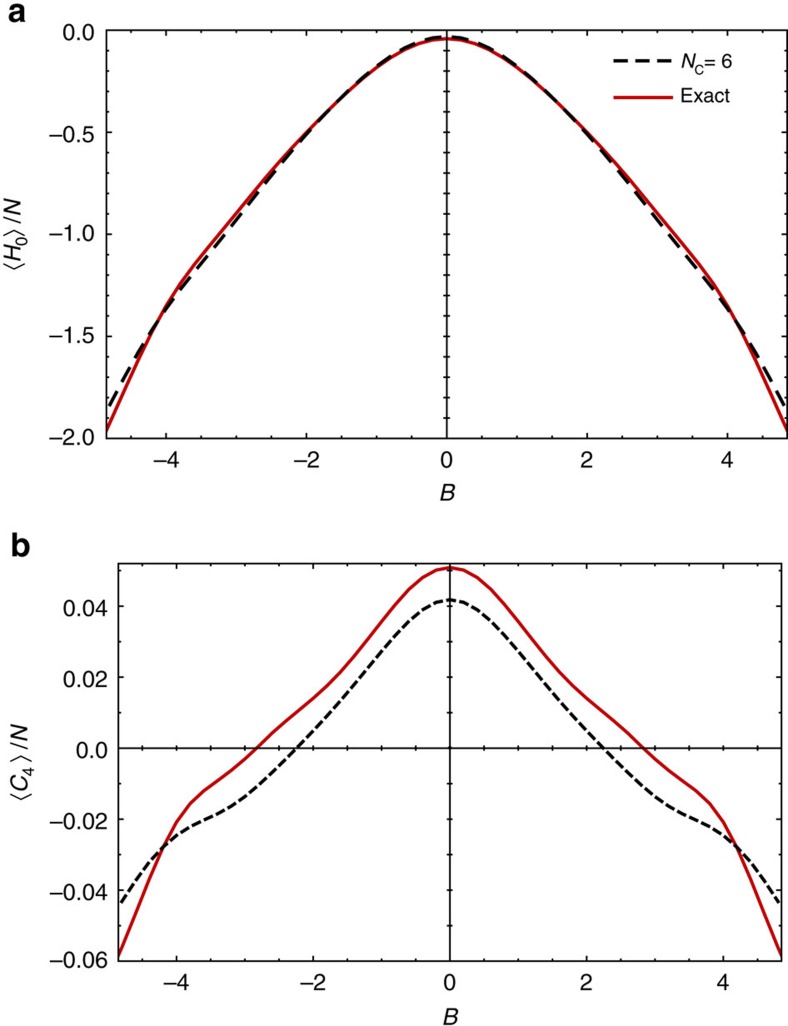
Additional verification of generalized Gibbs ensembles. (**a**) The energy density and (**b**) the expectation value of another conserved quantity *C*_4_ (4-spin operator) as a function of magnetic field *B*, obtained from calculation using all conserved quantities (solid) and a GGE with *N*_C_=6 (quasi-) local conserved quantities (dashed).

To provide further support for our claim that truncated GGEs give a semi-quantitative description of our weakly open system we show in Fig. 6 additional comparison of the 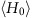 and 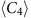 as a function of magnetic field *B* at 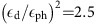, comparing as in the main text the exact calculation including all conserved quantities and the truncated GGE with *N*_C_=6 (quasi-)local conserved quantities. The GGE ansatz captures the right magnitude and the correct behaviour in the dependence on *B* also for more complicated 4-spin operators like *C*_4_. We use same parameters as for the Fig. 5 in the main text: 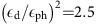, *J*=1, Δ=0.8, *ω*=1.6 *ω*_ph_, *ω*_ph_=*T*_ph_=1, *N*=12.

### Code availability

Custom computer codes used in this study are available from the corresponding author upon request. Documentation of the codes is not available.

### Data availability

Data is available from the corresponding author upon reasonable request.

## Additional information

**How to cite this article:** Lange, F. *et al*. Pumping approximately integrable systems. *Nat. Commun.* **8**, 15767 doi: 10.1038/ncomms15767 (2017).

**Publisher's note**: Springer Nature remains neutral with regard to jurisdictional claims in published maps and institutional affiliations.
